# Dacar Cas/Somali Red Aloe: a new species of *Aloe* (Asphodelaceae) from Somaliland

**DOI:** 10.3897/phytokeys.117.28226

**Published:** 2019-02-08

**Authors:** Mary E. Barkworth, Ahmed Ibrahim Awale, Faisal Jama Gelle

**Affiliations:** 1 Biodiversity Museum, University of Hargeisa, Hargeisa, Somaliland; 2 Biology Department, Utah State University, 5305 Old Main Hill, Logan Utah, 84322, USA; 3 Candlelight for Environment, Education, and Health, Hargeisa, Somaliland; 4 Faculty of Environmental Science, College of Applied and Natural Science, University of Hargeisa, Hargeisa, Somaliland

**Keywords:** Asparagales, species density, Africa

## Abstract

A new species of Aloe (Asphodelaceae) is described from Somaliland. It differs from other species in forming large clumps and in having sap that is initially yellow but quickly turns bright red and then dark red or reddish-brown, paniculate red-flowered inflorescences and uniformly coloured leaves with red teeth. Its recognition raises the number of species known from the combined area of Somaliland and Somalia s.s. from 31 to 36. A map portraying species density of *Aloe* by country, as that genus is now interpreted, shows that *Aloe* has its highest density on islands in the Indian Ocean but that, within Africa, the greatest density is in countries along the eastern highlands. The data also reinforce the importance of field botanists in determining a country’s known plant diversity.

## Introduction

The genus *Aloe* L. (*Asphodelaceae*) includes over 600 species, all native to Africa, islands in the western Indian Ocean or the Arabian Peninsula (Newton 2004; Carter et al. 2011; Klopper and Smith 2013). Its members are succulent plants with densely packed, spirally arranged leaves. They vary from stemless to having horizontal or vertical stems. The inflorescences vary from racemes to panicles with branches bearing spirally arranged pedicellate flowers that vary from being well-spaced to densely packed and distally concentrated. The flowers have a tubular perianth that is usually red to pink or scarlet but may be yellow, yellow-green or almost white. There are six stamens and a single gynoecium composed of three united carpels with many ovules. In most species, the fruits are capsules but a few form berries. Almost all species are considered to have medicinal and/or cosmetic value, but a few are poisonous. Only one, *A.vera* L., is widely cultivated. Because the species are often important to local people, there is considerable interest in the relationship among their use, distribution and phylogenetic relationships. This has resulted in publication of revisionary (Sebsebe and Nordal 2010; Klopper et al. 2012; Ruch et al. 2013; Klopper 2015; Cole and Forrest 2017) and phylogenetic (Grace et al. 2015) studies of the genus, plus development of an international database (Aloes of the World 2018) that enables easy visualisation of the distribution of the species richness by country.

Somaliland, which declared its independence from Somalia s.l. in 1991, lies along the south side of the Gulf of Aden. Its flora is treated in the "Flora of Somalia" (Thulin 1993, 1995, 1999, 2006) which was begun in 1988 (Thulin 1993, preface), before the breakdown of the central Somali government. In volume 4 of the "Flora of Somalia", Lavranos (1995) recognised 31 species of *Aloe*, all of them collected before 1990. In 1988, the unrest that eventually led to the breakdown of the central Somali government made botanical exploration challenging but, after its unilateral declaration of independence, Somaliland achieved a degree of peace and stability that, among other things, encouraged renewed exploration of its flora and fauna. This resulted in the addition of three previously undescribed species of *Aloe* to the country’s flora [*A.orlandi* Lavranos (Orlando 2003), *A.rubrodonta* T.A.McCoy & Lavranos and *A.kahinii* T.A.McCoy & Lavranos (McCoy and Lavranos 2007)] which were included in the appendices of the final two volumes of the flora to be published (Thulin 1999, 2006). A fourth species, *A.nugalensis* Thulin, was described later (Thulin 2012) based on plants grown from seeds collected in 1985. In this paper, we describe a fifth species, so far known only from Somaliland, locally known as “Dacar Cas” or “Red Aloe” but which we refer to, in English, as the Somali Red Aloe to distinguish it from the species currently associated with the English name “Red Aloe”, *A.cameronii* Hemsl. We also re-examined the distribution of *Aloe*, being interested in the extent to which recent discoveries have modified the information provided by Newton (2004).

## Materials and methods

**Figure 1.**
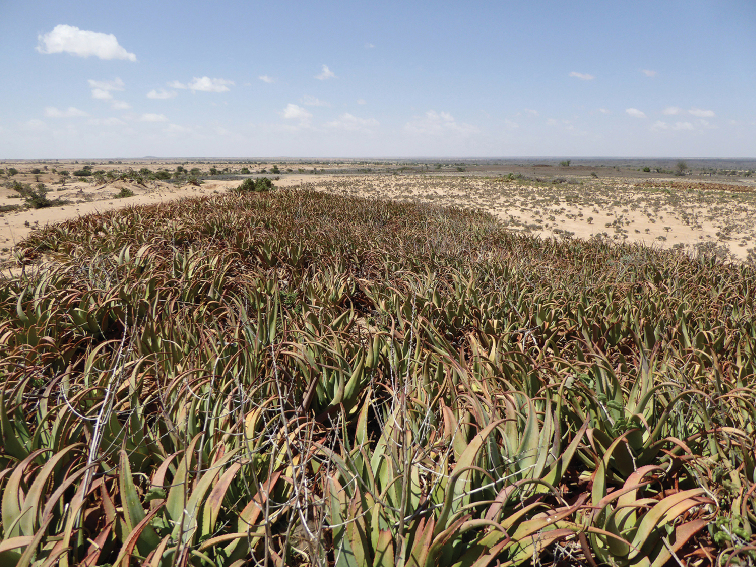
*Aloesanguinalis* at Alala Adka showing the largest clump in October 2016.

In 2014, Awale noticed an *Aloe* growing alongside a road near Alala Adka (Alaala Cadka) [Names are shown in English, followed by the Somali name in parentheses], Marodi Jeh (Maroodi Jeex) Region, Somaliland, that was unlike other native species in the area in forming large, dense patches (Fig. 1). They also differed from other *Aloe* species in the region in having leaves with reddish teeth and, when cut, an exudate that rapidly turns from yellow to bright red. Local people had also noticed the plants, referring to them as “Da’ar As (Dacar Cas), or Red Aloe, to distinguish them from other Aloes in the region, such as Da’ar Buduk (Dacar Budhuq), the name used locally for *A.retrospiciens* Reynolds.

In October 2016, Awale and Barkworth visited the Alala Adka site to take photographs and make measurements. The location is a treeless plain in the Oogo ecological zone (Ibrahim 2010), dominated by shrubs and showing signs of overgrazing (Fig. 1). Unfortunately, no flowers were present. Despite this, they attempted to determine the scientific name of the plants using "Flora of Somalia" (Lavranos 1995; Thulin 1999, 2006) but none of the descriptions fitted, nor did those of other publications consulted, such as Ruch et al. (2013). They sent images of the plants to Mats Thulin, the late John Lavranos and Tom McCoy, three individuals highly respected for their knowledge of the region’s flora, Lavranos and McCoy being the second and third most prolific describers of *Aloe* species (Klopper and Smith 2013). All stated that the images sent were not of a species with which they were familiar and advised returning when the plants were in flower, partly to enable preparation of a complete description, but also to investigate the possibility that the plants were hybrids.

**Figure 2.**
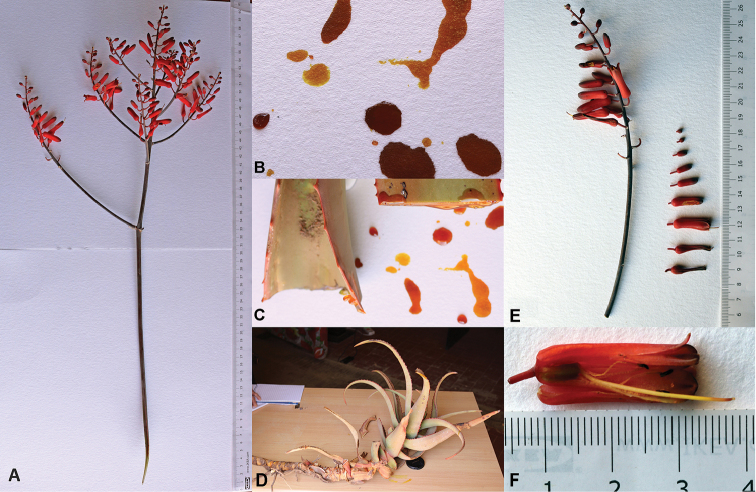
*Aloesanguinalis* from Alala Adka, June 2017. **A** Whole inflorescence, including peduncle **B** Sap, showing color after 10–15 minutes **C** Leaves freshly cut near their base showing color of sap when fresh **D** Decumbent offset, showing roots from some nodes **E** Inflorescence branch and flowers from another branch, arranged from top to bottom of the branch **F** Flower after maturation of style.

In May 2017, Awale and Barkworth revisited the site and found flowers. They prepared specimens, recorded measurements and took photographs both in the field and in the herbarium (Fig. 2). Awale also collected cuttings to grow and observe in Hargeisa. The flowers were protandrous, forming plump, well-filled anthers that matured before the styles had fully elongated. Both the anthers and the styles appeared fully functional. These observations made it unlikely that the plants were hybrids.

**Figure 3.**
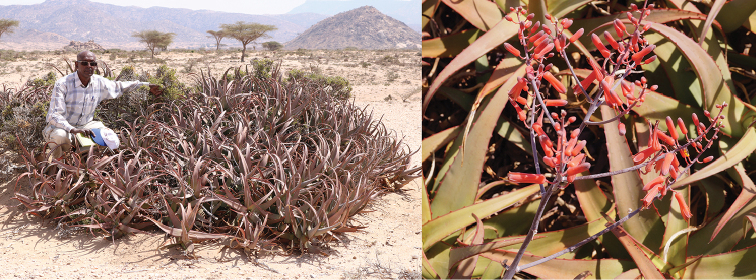
*Aloesanguinalis* at Lafarug. **A** One of the larger clumps, with Awale, in January 2018 **B** Inflorescence of *Aloesanguinalis* at Lafarug, June 2018.

In January 2018, Awale discovered another population growing near Lafarug (Lafaruug), in Sahil (Saaxil) Region, alongside the road to Hiin-Weyne village. Later that month, Barkworth and Awale revisited the location to learn more about the plants there and to make measurements. Lafarug is in the subcoastal area below the Golis escarpment and has sandy soil with semi-desert vegetation that includes *Vachelliatortilis* (Forssk.) Galasso & Banfi, *Doberaglabra* (Forssk.) Poir., *Salvadorapersica* L., *Indigoferasparteola* Chiov. and *Commiphora* Jacq. (Fig. 3A). There were no flowering inflorescences in January but some old ones were collected and examined for seeds. Awale revisited the site in June 2018, at which time the plants were in flower (Fig. 3B), and again in August 2018. During the August trip, he collected seeds.

**Figure 4.**
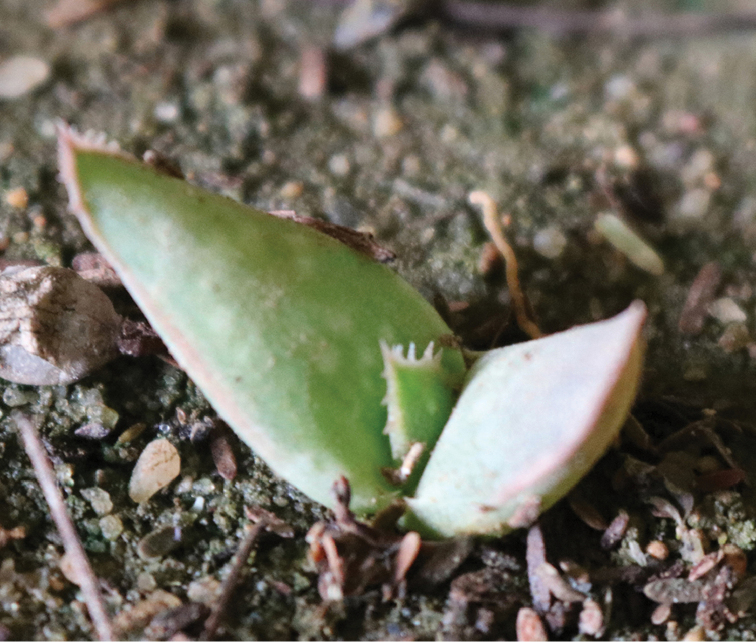
Seedling of *Aloesanguinalis*, grown from seeds collected at Lafarug in August 2018.

Most capsules collected in January at Alala Adka had no seeds; none had more than 3. Capsules collected from the Lafarug population in August 2018 had several seeds. A total of about 50 seeds from the Lafarug area were planted in a pot and kept moist. By November 2018, 12 had germinated and one had developed three leaves (Fig. 4).

Based on the comments by Thulin, Lavranos and McCoy and the apparent functionality of the anthers and styles, we proposed recognising the Somali Red Aloe as a new species in July but, at the urging of the reviewers, Barkworth reviewed all the *Aloe* images made available by the Royal Botanical Gardens, Kew (Kew 2018), specimens in the herbarium of the Missouri Botanical Garden and, together with Gelle, those in the East African Herbarium of National Museums, Kenya. Because many of the distinguishing features of Aloes are poorly preserved in herbarium specimens, we also reviewed the species descriptions for specimens that could not, based on the herbarium specimens seen, readily be distinguished from the Somali Red Aloe. These studies supported our earlier conclusion that the Somali Red Aloe should be described as a new species. In preparing the description, we have used terminology that is consistent with Klopper et al. (2013) but avoided use of the term “raceme” because of inconsistencies in how it is used [compare, for example, Klopper et al. 2013 with Harris and Harris (2001) and Beentje (2016)].

To examine current knowledge about the distribution of species richness in Aloe, we updated Newton’s table with data from multiple sources [(Reynolds 1954; Klopper et al. 2012; Ruch et al. 2013; Rakotoarisoa et al. 2014; Klopper 2015; Rakotoarisoa and Grace 2017; Cole and Forrest 2017; Aloes of the World (2018)] and calculated the number of species per km^2^. In doing so, we used the current interpretation of *Aloe* which excludes *Aloidendron* (A. Berger) Klopper & Gideon F. Sm., *Aloiampelos* Klopper & Gideon F. Sm. and other small genera previously included in it.

## Taxonomic treatment

### Aloe sanguinalis

Taxon classificationPlantaeAsparagalesAsphodelaceae

Awale & Barkworth sp. nov.

urn:lsid:ipni.org:names:60478025-2

Figures 1 , 2 , 3 , 4

#### Type.

SOMALILAND. Marodi Jeh (Maroodi Jeex), Hargeysa, Alala Adka, 15–20 km west of the town of Da’ar Buduk (Dacar Budhuq). Elevation 950 m, 9.8705N, 44.3761E (WGS84), 24 May 2017, *Mary E. Barkworth S17.001, Ahmed Ibrahim Awale, Garrett Billings* and *Helen Pickering* (holotype: HARG).

#### Diagnosis.

*Aloesanguinalis* differs from other species of *Aloe* in its combination of sap that is initially yellow but quickly turns bright red, drying to dark red or brownish-red, strong clump-forming habit, red teeth and paniculate inflorescence of well-spaced glabrous, red flowers.

#### Description.

Plants with decumbent stems, rooting at the lower nodes, forming clumps 1–10(–40) m long in their longest direction; stems leafy, the terminal 50–100 cm vertical. Leaves 30–40 cm long, 5–8 cm wide at the base, lanceolate, evenly blue-green on both surfaces when young, becoming suffused with reddish colouration at the margins and the distal portion of their upper surfaces with age, crescent-shaped in cross-section when dry; margins with sharp red teeth 4–6 mm long spaced 1–3 cm apart near the base of the leaves, closer together towards the leaf tips; sap yellow when fresh, rapidly becoming bright red, drying to dark red or brownish-red, without a noticeable odour. Inflorescences paniculate, 70–120 cm long (including peduncle), 20–75 cm long (excluding peduncle), lower primary branches (15–)23–50 cm long, ascending to arcuate, often with 1(–2) secondary branches, flowers confined to the distal 1/2–3/4 of the branches, pedicellate and spirally arranged, not secund, distance between lowest flowers 5–11 mm; bracts subtending the pedicels 5–9 mm long, narrowly triangular; pedicels about 10 mm long, not elongating in fruit; perianths (including lobes) 20–25 mm long, red, glabrous; outer perianth lobes 10–15 mm long, slightly recurved distally, with narrow, hyaline, minutely erose margins; stamens 6, the anthers versatile, ca. 2 mm long, extending about 1 mm beyond the perianth at anthesis but the perianth extending 3–5 mm beyond the anthers as the style matures; ovary superior, with many ovules, stigma exserted 5–9 mm beyond the perianth at maturity. Capsules 15–20(–25 mm) long, 13–20 in diameter, ovoid, woody; seeds winged, 5–11 mm across (including the papery wing), triangular-pyramidal, outer surfaces ca. 1 mm across.

#### Distinguishing features.

*Aloesanguinalis* caught Awale’s attention by the large size of its clumps but its most distinctive feature appears to be the colour of its sap (Fig. 2) which is yellow, quickly turning bright red, then drying dark red or brownish-red. The difficulty is that none of the descriptions in the "Flora of Somalia" refer to sap colour. Carter et al. (2011) mention sap features, usually colour, but for only 186 of the 517 species treated. In most species, the colour is described as some variant of yellow, but *A.rabaiensis* Rendle has sap described as “yellow, drying red” and *A.volkensii*, sap described as “yellow, drying dark reddish”. *Aloerabaiensis* is a shrubby aloe which differs from *A.sanguinalis* in having flowers in “sub-capitate to capitate” clusters (Carter et al. 2011, p. 633). It extends from Tanzania to southern Somalia s.s. *Aloevolkensii* Engl. is a tree aloe described as having densely flowered, “subcapitate to cylindrical-conical” flower clusters. It is known from south-eastern Kenya and north-eastern Tanzania (Carter et al. 2011, p. 691).

There were 16 other species described as having sap that dries purplish or reddish-brown. Of them, none is known from Somaliland, but *A.gillettii* S. Carter grows in Puntland, Somalia “on limestone with *Juniperus* in open *Commiphora* bushland” (Lavranos 1995, p. 40), a different habitat from that of *A.sanguinalis*. *Aloegillettii* also differs from *A.sanguinalis* in having “dark grey-green leaves with very numerous white spots” and “white, cartilaginous teeth to 1 mm long” (loc. sic). The other species would also be ruled out by the description given.

There are other clump-forming species in Somaliland and Somalia but none, so far as we are aware, that forms such large clumps. Again, the difficulty is that existing descriptions merely refer to forming small or large clumps.

#### Distribution.

*Aloesanguinalis* is currently known from only two locations, the type locality near Alala Adka and a more northern locality at 9.9840N, 44.8195E near the village of Lafarug. Larajasse (1897, p. 25), a Catholic missionary based in Berbera from 1888–1903, stated that “da’ar” refers to bile or an “aloe about three feet high, red and orange varieties, broad spiked fleshy leaves, spreading out from the ground; is a favorite food of elephants.” It seems probable, considering the species known from the area, that he was referring to *A.sanguinalis*. Elephants have not been seen in Somaliland since 1958.

#### Habitat and ecology.

The two known locations of *Aloesanguinalis* are open plains with sandy soils in which, among other species, *Salvadorapersica* and *Indigoferasparteola* grow. The Alala Adka location is treeless but there are scattered *Vachelliatortilis* trees at the Lafarug site.

#### Phenology.

Flowering time in *Aloesanguinalis* is probably determined by the timing of the spring rains which fall between late March and early June. The optimum time for seed collection has not been determined, but it is likely to be July to September.

#### Etymology.

The epithet is derived from *sanguineus*, Latin for blood, and refers to the colour of the sap which distinguishes it from all other species in the region.

## Comments

### Conservation status

The two known populations of *Aloesanguinalis* do not appear to be under immediate threat. Both areas are used for livestock (goats, camels) grazing but there was no indication that *A.sanguinalis* was being eaten, nor was there any indication that leaves were being over-harvested. Some clumps appear to have been split in two, possibly by grazing animals. Once a path through a clump is established, it is likely to become permanent as the soil will be compacted making seedling and offset establishment more difficult.

At both Alala Adka and Lafarug, there are multiple clumps of *Aloesanguinalis*. There were some markings on the leaves suggesting that insects might be piercing their surface but otherwise, the plants seemed undamaged. All the plants appeared to be growing on slight mounds in the sandy soil, probably because they protect the soil from being blown away. Offsets from both sites have been planted successfully in Hargeisa.

The largest clumps of *Aloesanguinalis* were those at the first site, Alala Adka, the largest being about 40 m long and 10 m wide. We estimated that it was composed of about 2500 stems. The clumps at Lafarug were considerably smaller, none that we saw being more than 5 m long in its longest dimension. We do not know what proportion of the vertical stems within a clump represents new individuals rather than established offsets. It seems probable, given the obstacles drought and grazing present to seedlings, that most growth is vegetative. Time constraints precluded a detailed survey of the two sites.

The biggest threat to the species may be the increasing frequency of drought which makes establishment of new plants, whether from seed or offsets, difficult. We saw no seedlings at either location but did not conduct a deliberate search for them within the clumps nor underneath other shrubby vegetation, the locations where they are most likely to become established. Because so little is known about the distribution, phenology, pollination, seed set and reproductive success of *Aloesanguinalis*, we recommend it be regarded as “data deficient”.

### Herbarium studies

*Aloe* tends to make very poor herbarium specimens. Both leaves and flowers tend to lose their colour, making it essentially impossible to tell from most specimens whether, in life, the leaves were evenly coloured and what colour the leaf margins and teeth had been. Moreover, in many instances, the preserved leaves were much smaller than indicated in the descriptions, collectors tending to collect leaves, or portions of leaves, to fit herbarium sheets. In a few instances, the label contained colour information and notes about the size of the plants and the leaves, but such specimens were exceptional. Despite these restrictions, the images provided by the Royal Botanic Gardens, Kew (Kew 2018) were invaluable. Unfortunately, records from the other herbaria likely to have substantial holdings of *Aloe* from Somaliland and other parts of the Horn of Africa (EA, ETH and UPS; codes from Thiers 2018) are not yet available on line. In future, we encourage individuals collecting aloes to supplement their specimens with images of the living plants, a suggestion that digital technology has made feasible, and that herbaria mobilise their specimen records using software (for example, Symbiota 2018) that can accommodate multiple images per specimen.

### Distribution of species density

**Figure 5.**
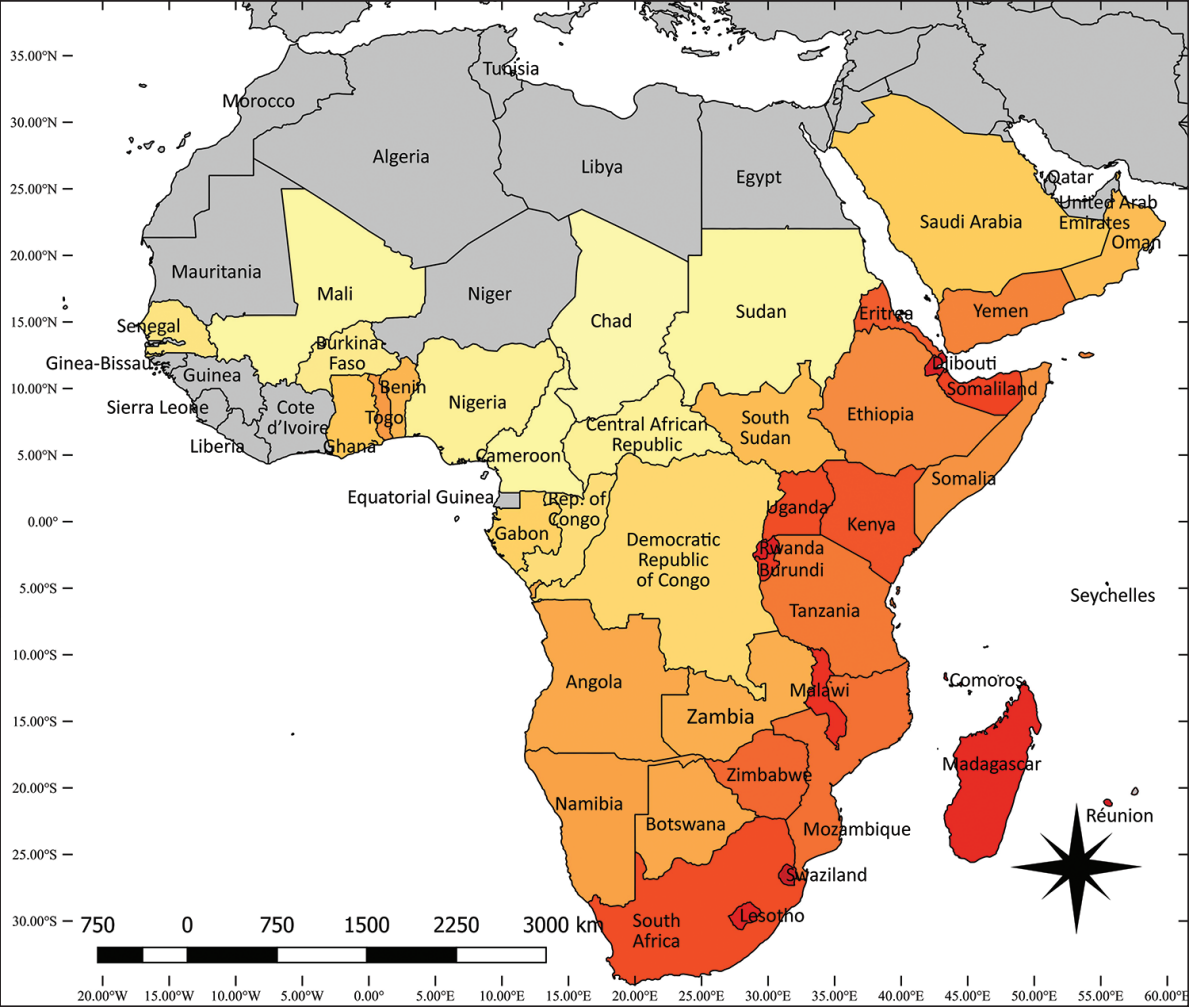
*Aloe* species density by country. Lowest density pale yellow (Chad), deep red (as in Réunion); highest densities are for islands too small to show on map; grey – no species known. See Table 1 for data.

Updated information on the number of *Aloe* species present in different countries (Table 1) shows 96 more records than in Newton (2004). although the numbers for a few countries have decreased, probably because of the exclusion of *Aloidendron*, *Aloiampelos* and a few other small genera. The overall increase reflects both recognition of new species and the discovery of known species in additional countries. The highest species densities (Table 1, Fig. 5) are on ocean islands and in countries that are so small that, even if only 1 species is present, the species density is high. Within Africa, the highest species densities belong to countries of the eastern highlands and coast. The general distribution of species richness is similar to that in Grace et al. (2015), in which the information is related to the genus’ phylogenetic history.

**Table 1.** *Aloe* L. species richness and density. ND: no data.

Country or Area	Area in km^2^	Species Newton (2004)	Species Current	Species Density (species/km^2^)
Aldabra	155	1	1	0.006435
Angola	1246799	24	29	0.000023
Benin	110622	3	2	0.000018
Botswana	566730	8	13	0.000023
Burkina Faso	273800	1	1	0.000004
Burundi	25680	1	5	0.000195
Cameroon	472710	1	1	0.000002
Central Africa Republic	622984	ND	2	0.000003
Chad	1259200	ND	1	0.000001
Comoros	2235	1	2	0.000895
Djibouti	23180	ND	7	0.000302
Democratic Republic of Congo	2267048	13	15	0.000007
Eritrea	101000	8	10	0.000099
Ethiopia	1000000	34	45	0.000045
Gabon	257667	ND	2	0.000008
Ghana	227533	3	3	0.000013
Côte d’Ivoire	318003	ND	1	0.000003
Kenya	569140	55	63	0.000111
Lesotho	30355	8	8	0.000264
Madagascar	581540	77	129	0.000222
Malawi	94080	17	19	0.000202
Mali	1220190	3	3	0.000002
Mauritius	2030	2	3	0.001478
Mozambique	780380	25	45	0.000058
Namibia	823290	26	24	0.000029
Nigeria	910768	3	3	0.000003
Oman	309500	5	5	0.000016
Pemba	988	2	2	0.002024
Republic of Congo	341500	ND	2	0.000006
Réunion	2512	1	1	0.000398
Rodrigues	108	1	1	0.009259
Rwanda	24668	4	7	0.000284
Saudi Arabia	2149690	22	23	0.000011
Senegal	192530	ND	1	0.000005
Seychelles	455	1	3	0.006593
Socotra	3665	3	5	0.001364
Somalia s.s.	451217	17	19	0.000042
Somaliland	176120	23	27	0.000153
South Africa	1214470	119	136	0.000112
South Sudan	644329	ND	10	0.000016
Sudan	1861484	ND	4	0.000002
Swaziland	17204	18	29	0.001686
Tanzania	885800	40	50	0.000056
Togo	54385	1	2	0.000037
Uganda	197100	16	22	0.000112
Yemen	527968	26	29	0.000055
Zambia	743398	19	16	0.000022
Zanzibar	1464	2	1	0.000683
Zimbabwe	386847	27	33	0.000085

Table 1 and the citations used to update it demonstrate that aloes are more likely to be discovered in countries where they are the focus of field botanists fascinated by the genus. This point was brought out even more clearly by Crouch et al. (2013) in their analysis of species discovery, collecting effort and collectors in southern Africa. They conclude that “Further new descriptions are likely to flow from active fieldwork in rugged environments.” (Crouch et al. 2013 p. 195). We agree concerning the higher probability of making new discoveries in rugged environments but, as this paper and that describing *A.orlandi* (Lavranos 2006) demonstrate, there are still discoveries to be made in easily accessible habitats. To facilitate research on Somaliland’s species, the University of Hargeisa and Somaliland Biodiversity Foundation have established an *ex situ* conservation site in front of the university’s Biodiversity Museum. Those interested in collecting aloes and other species in Somaliland should contact the Ministry of Environment and Rural Development to obtain a collecting permit before applying for a visa and well in advance of their planned visit.

## Supplementary Material

XML Treatment for Aloe sanguinalis
